# Combining multimodal imaging and treatment features improves machine learning‐based prognostic assessment in patients with glioblastoma multiforme

**DOI:** 10.1002/cam4.1908

**Published:** 2018-12-18

**Authors:** Jan C. Peeken, Tatyana Goldberg, Thomas Pyka, Michael Bernhofer, Benedikt Wiestler, Kerstin A. Kessel, Pouya D. Tafti, Fridtjof Nüsslin, Andreas E. Braun, Claus Zimmer, Burkhard Rost, Stephanie E. Combs

**Affiliations:** ^1^ Department of Radiation Oncology Klinikum rechts der Isar der Technischem Universität München (TUM) München Germany; ^2^ Deutsches Konsortium für Translationale Krebsforschung (DKTK), Partner Site Munich Munich Germany; ^3^ Department of Radiation Sciences (DRS), Institute of Innovative Radiotherapy (iRT) Helmholtz Zentrum München Neuherberg Germany; ^4^ Allianz SE Munich Germany; ^5^ Department of Nuclear Medicine Klinikum rechts der Isar der Technischen Universität München (TUM) Munich Germany; ^6^ Department for Bioinformatics and Computational Biology Technical University of Munich (TUM) Garching Germany; ^7^ Department of Neuroradiology Klinikum rechts der Isar der Technischen Universität, Munich (TUM) München Germany

**Keywords:** biomarker, FET‐PET, glioblastoma, machine learning, MRI, prognostic model, VASARI

## Abstract

**Background:**

For Glioblastoma (GBM), various prognostic nomograms have been proposed. This study aims to evaluate machine learning models to predict patients' overall survival (OS) and progression‐free survival (PFS) on the basis of clinical, pathological, semantic MRI‐based, and FET‐PET/CT‐derived information. Finally, the value of adding treatment features was evaluated.

**Methods:**

One hundred and eighty‐nine patients were retrospectively analyzed. We assessed clinical, pathological, and treatment information. The VASARI set of semantic imaging features was determined on MRIs. Metabolic information was retained from preoperative FET‐PET/CT images. We generated multiple random survival forest prediction models on a patient training set and performed internal validation. Single feature class models were created including "clinical," "pathological," "MRI‐based," and "FET‐PET/CT‐based" models, as well as combinations. Treatment features were combined with all other features.

**Results:**

Of all single feature class models, the MRI‐based model had the highest prediction performance on the validation set for OS (C‐index: 0.61 [95% confidence interval: 0.51‐0.72]) and PFS (C‐index: 0.61 [0.50‐0.72]). The combination of all features did increase performance above all single feature class models up to C‐indices of 0.70 (0.59‐0.84) and 0.68 (0.57‐0.78) for OS and PFS, respectively. Adding treatment information further increased prognostic performance up to C‐indices of 0.73 (0.62‐0.84) and 0.71 (0.60‐0.81) on the validation set for OS and PFS, respectively, allowing significant stratification of patient groups for OS.

**Conclusions:**

MRI‐based features were the most relevant feature class for prognostic assessment. Combining clinical, pathological, and imaging information increased predictive power for OS and PFS. A further increase was achieved by adding treatment features.

## 1 INTRODUCTION

Glioblastoma multiforme (GBM) constitutes the most frequent primary neuronal malignancy. Despite intensive efforts in research, the success of current therapy regimens remains limited with low two‐year survival rates of 16.9%.^1^ We know that clinical parameters such as younger age, high Karnofsky‐performing status (KPS) indices, and female gender correlate with a favorable outcome.^2^, ^3^, ^4^, ^5^, ^6^, ^7^, ^8^ Several approaches finding molecular determinants of outcome to predict treatment response have been published. However, few have reached clinical relevance.^9^, ^10^, ^11^ MGMT‐promoter methylation status and mutational status of IDH appear to carry prognostic value.^11^, ^12^, ^13^, ^14^

MRI constitutes the standard imaging modality for pre‐therapeutic staging, treatment planning and follow‐up diagnostics. Over the years, multiple prognostic relevant semantic properties have been identified. Proposed features quantify the different composites of the tumor (eg, enhancement or edema), or classify characteristics such as multifocality or invasion of brain areas.^3^, ^4^, ^15^, ^16^, ^17^ MRI‐based quantification of the extent of resection emerged as prognostic factor.^2^, ^18^

Based on such qualities, the VASARI (Visually Accessible REMBRANDT [Repository for Molecular Brain Neoplasia Data] Images) feature list was defined by the REMBRANDT consortium aiming to standardize the reporting of gliomas. Inter‐observer agreement appeared to be high in all but three imaging features.^19^ Multiple publications have shown that VASARI features predict patient outcome, and correlate with mutational status and gene expression patterns.^20^, ^21^, ^22^

In recent years, metabolic PET images utilizing amino acid‐based tracers gained clinical relevance. For instance, parameters obtained from static [18F]‐fluoroethyl‐l‐tyrosine (FET) PET/CT were shown to inherit prognostic value predicting survival and progression independent of MGMT promoter methylation and clinical factors.^23^ Moreover, FET uptake variables significantly correlated with WHO grading.^24^, ^25^

Originally, prognostic models aiming at predicting patients' survival or progression were often based on statistical models. For example, a recently published model by Gittleman et al^26^ predicted survival on the basis of age, gender, MGMT‐promoter methylation status, and KPS. In recent years, machine learning (ML) approaches have gained significant importance as alternative way for model generation. Due to the improvements in handling large datasets with many input features, ML‐based methods may lead the way to clinical decision support systems (CDSS) as the basis for personalized medicine.^27^

In the present study, we sought to determine the prognostic value of ML‐based models on the basis of multiple input feature classes including clinical, pathological, semantic MRI features, and FET‐PET/CT measurements. Moreover, we analyzed if "multimodal" models combining several feature classes and, specifically, the addition of treatment features further improve the prognostic performance of ML models.

## 2 MATERIAL AND METHODS

### 2.1 Patients and study design

In total, 189 patients with GBM treated with radiation therapy (RT) from 2009 to 2016 in our institution were retrospectively analyzed. Patients received established first‐line therapy after informed consent following the primary diagnosis. Patient records were assessed for gender, age, and KPS at start of RT (see Table 1). Pathological records were assessed for MGMT‐promoter methylation status, IDH1 mutation status, and the KI67 proliferation index. Immunohistochemistry using an antibody against the IDH1 p.R132H mutation was used to test for IDH1 mutations. The MIB1 antibody was used to test for the KI67 proliferation index. The Ki67 proliferation index was split at the median of 20% dividing high and low proliferation groups. Promoter methylation was determined using real‐time PCR‐based methylation‐quantification of endonuclease‐resistant DNA (MethyQESD) method.^28^ MGMT promoter methylation was defined by promoter methylation greater than 8% as described by Reifenberger et al.^13^

**Table 1:** Patient characteristics and outcome

	Training set (n = 132)	Validation set (n = 57)
Age (y)	m 58 (min 20, max 85)	m 59 (min 24, max 82)
KPS (%)	m 80 (min 40, max 100)	m 80 (min 40, max 100)
Gender	Male: 82 Female: 50	Male: 39 Female: 18
Positive MGMT‐methylation status	33 (25%) (na: 38 [29%])	13 (23%) (na: 16 [32%])
Positive IDH1/2 mutation status	3 (3%) (na: 55 [42%])	1 (5%) (na: 21 [37%])
Ki‐67 proliferation index	>20%: 32 (24%) <20%: 31 (24%) (na:69 [52%])	>20%: 16 (28%) <20%: 13 (23%) (na: 28 [49%])
OS (mo)	m 11.9 (min 0, max 75.5)	m 11.7 (min 0.3, max 87.4)
PFS (mo)	m 4.25 (min 0, max 60.8)	m 6.3 (min 0, max 74.0)
Preoperative MRI	124 (94%)	54 (95%)
Postoperative MRI	99 (75%)	46 (81%)

KPS, Karnofsky performance status; m, median; max, maximum; min, minimum; na, not available; OS, overall survival; PFS, progression‐free survival.

Primary tumor resection with sequential radio(chemo)therapy constituted the first line therapy, which was possible in 162 patients. 27 patients were primarily treated with RT after biopsy. 149 patients received concomitant radiochemotherapy following the protocol of Stupp et al^11^ with radiotherapy (RT) up to a total dose of 60 Gy (single dose 2 Gy) and temozolomide (75 mg/m^2^) (see Table 2 for therapy characteristics). In addition to temozolomide, one patient received cilengitide and two patients received lomustin. One patient received bevacizumab and three patients were additionally treated with irinotecan.

**Table 2:** Therapy characteristics

	Training set (n = 132)	Validation set (n = 57)
Primary Surgery	112 (85%)	50 (88%)
Primary Radiation	20 (15%)	7 (12%)
RTCT	Yes: 103 (78%) No: 8 (6%) (na: 21 [16%])	Yes: 45 (79%) No: 3 (5%) (na: 9 [16%])
Adjuvant CT	Yes: 88 (67%) No: 13 (10%) (na: 31 [23%])	Yes: 41 (72%) No: 4 (7%) (na: 12 [21%])
Therapeutic Interval>6 weeks[Link]	Yes: 32 (24%) No: 79 (60%) (na: 21 [16%])	Yes: 37 (65%) No: 13 (23%) (na: 7 [12%])
PTV (mL)	m: 333.9 (min 29.9, max 701) (na: 4 [3])	m: 340.8 (min 57.9, max 862.4) (na: 1 [2%])
TD (Gy)	m 60 (min 10, max 64)	m 60 (min 18, max 60)
SD (Gy)	m 2.0 (min 1.8, max 5.0)	m 2.0 (min 1.7, max 3.0) (na: 2 [4%])

CT, chemotherapy; m, median; max, maximum; min, minimum; na, not available; PTV, planning target volume; RT, radiotherapy; RTCT, radiochemotherapy; SD, single dose; TD, total dose.

Between surgery and RT.

Overall survival (OS) was determined from the end of RT to the time point of death or the time point of censoring (134 reported deaths). Progression‐free survival (PFS) was calculated from the end of RT to the first sign of progression, death or time point of censoring (168 reported progressions or deaths), whichever happened first. Progress was defined retrospectively according to MRI‐ and PET‐study reports and/or pathological reports. All clinical and molecular data were collected in the Munich Innovative Radiotherapy (MIRO) database. This study was approved by the ethical committee of the Technical University of Munich (reference number 466/16).

### 2.2 Magnetic resonance imaging

Pre‐ and postoperative MRIs were assessed for availability of relevant sequences: T1‐weight (T1w)‐ (or MPRAGE), T1w+Gd‐, FLAIR‐, T2w‐ and diffusion imaging with apparent diffusion coefficient (ADC)‐maps (see Table S1 for image acquisition parameters). All 27 preoperative features and three postoperative features were determined following the recommendations of the REMBRANDT consortium by a MD with 2 years of experience in radiation oncology (see Table S2 for all VASARI features).^19^ The ADC‐maps were used to classify "facilitated," "restricted," and "mixed" diffusion. Before analysis, few features were altered to achieve a better patient representation in subgroups (eg, pooling of subgroups) or to retain a binary variable (see Table S3).

### 2.3 [18F]‐fluoroethyl‐l‐tyrosine (FET) PET studies and analysis

FET‐PET/CT scans were performed pre‐operatively using a Biograph 16 PET/CT in 68 patients (Siemens Medical Solutions USA, Inc., Malvern, PA, USA). Patients were required to fast for a minimum of 6 hours before undergoing scanning to achieve standardized metabolic conditions. 190 MBq of FET were intravenously administered and a low‐dose CT (24‐26 mAs, 120 kV) for attenuation correction was conducted. 30‐40 minutes after initial injection, the 10‐minute PET acquisitions were performed. Static PET data were reconstructed by two different methods. PET/CTs of 49 patients were reconstructed using filtered back‐projection employing a Hann filter with a cut‐off frequency of 0.34 Nyquist into 128 × 128 matrices, with a resulting voxel size of 2.1‐2.1 mm and slice thickness of 2.4 mm. In 19 patients, an ordered subset expectation maximization algorithm was used (200 × 200 matrix, 3 iterations, 21 subsets, resulting in a voxel size of 1.6 to 1.6 mm).

Semiautomatic analysis of static FET‐PET was performed by an experienced nuclear medicine physician (PT), blinded to histology and clinical outcome, using Matlab (MathWorks, Inc., Natick, MA, USA; Image Processing Toolbox and own code). The procedure was performed as described earlier.^25^ Briefly, the images were normalized against background uptake, defined with a region of interest placed into the hemisphere opposite to the tumor as proposed by the German guidelines for brain tumor imaging.^29^ Tumor segmentation was conducted by placing seed‐points inside the tumor followed by automated region‐growing, which was limited, by a margin of 1.3 times the background activity. If necessary, blocking lines were placed manually to prevent the algorithm from growing into surrounding anatomic structures with increased PET signal. Maximum tumor to brain ratio (TBR), mean TBR, metabolic tumor volume (MTV), and the product of mean TBR and MTV, which was defined as total lesion normalized uptake (TLU), were calculated on floating‐point data (see data in Table S4).

### 2.4 Building of ML models

Machine learning modeling and statistical analyses were performed in R (version 3.4.0) (R core team, Vienna, Austria).For all testing purposes, the data set for each of the seven prediction models was randomly split into one development subset containing 2/3 of all patients (n = 132) and one independent test subset containing 1/3 of all patients (n = 57). No data from the independent test subset were used for the development of Models 1‐7. As ML technique, the random forest algorithm, implemented as an ensemble of decision trees constructed from randomly selected features and training data points, was chosen due to its short training periods, the capability of managing incomplete and noisy data, good interpretability, and high predictive power.^30^, ^31^ To predict right censored survival outcomes random survival forest (RSF) models were developed using the *randomForestSRC* package (R core team).^32^ The VIMP function was applied to calculate feature permutation importance. Altogether seven prediction models on the basis of different feature classes were trained (see Table 3 for selected features): Firstly, four models were generated on single feature classes including "clinical" (model 1 (M1)), "pathological" (M2), "MRI‐based" (M3), and "FET‐PET/CT‐based" (M4). Secondly, the benefit of combining clinical and pathological features (M5) and all four feature classes combined (M6) was tested. Finally, treatment features were added to all four feature classes (M7).

**Table 3:** Machine learning models with multimodal feature classes

Model and feature class	Features
M1: "Clinical"	Age, KPS, Gender
M2: "Pathological"	MGMT‐promoter‐methylation, IDH‐mutational status, Ki 67%‐PI
M3: "MRI‐based"	VASARI features
M4: "FET PET/CT‐based"	TBRmax, TBRmean, MTV, TLU
M5: "Clinical/Pathological"	M1 + M2
M6: "Clinical/Pathological/Imaging"	M1 + M2 + M3 + M4
M7: "Clinical/Pathological/Imaging" + treatment features	M1 + M2 + M3 + M4 + RTCT, surgery, PTV, TD, SD, adjuvant CT, therapeutic Interval[Link]

CT: chemotherapy; KPS: Karnofsky performance status; MTV: mean tumor volume; PI: proliferation index; PTV: planning target volume; RT: radiotherapy; RTCT: radiochemotherapy; SD: single dose; TBR: tumor to brain ratio; TD: total dose; TLU: total lesion uptake.

Between surgery and RT.

### 2.5 Performance evaluation

The performance was assessed on the independent patient test set. The concordance index (C‐index) served as performance estimator. Direct comparison of models was performed using the rcorr.cens function of the *Hmisc* package. For dichotomization of patient subgroups, the maximally selected rank statistics method was applied to the training set to determine the optimal cut‐off value using the *maxstat* package.^33^ The same cut‐off value was then used to define high‐risk and low‐risk patients in the validation set. Log‐rank tests were conducted to test for statistical significance between patient risk groups. The area under the receiver operator characteristic curve (AUC) was calculated using the *survivalroc* package.

## 3 RESULTS

### 3.1 Prediction of patients' OS

First, the predictive value of ML models based on single variable classes was tested for OS (see Table 4 for C‐index values and confidence intervals of all models). The clinical (M1) and pathological (M2) models showed low predictive capability in both development (C‐index: M1 0.74 [95% confidence interval: 0.67‐0.81], M2: 0.64 [0.58‐0.71]) and independent test set with no significant difference from random in the latter (C‐index: M1 0.59 [0.48‐0.70], M2: 0.49 [0.37‐0.60]). With a C‐index of 0.93 (0.87‐1.00), the MRI‐based model (M3) had the highest prediction performance on the development set, which could be validated, with a reduced C‐index of 0.61 (0.51‐0.72) significantly different from random. The FET‐PET/CT‐based model (M4) showed high prediction capacity on the development set (C‐index: 0.93 [0.86‐1.00]), which was, however, not reproducible on the validation set (C‐index: 0.54 (0.44‐0.65)).

**Table 4:** Performance estimates as concordance index (C‐index) and 95% confidence intervals of all seven models predicting OS and PFS

		M1	M2	M3	M4	M5	M6	M7
OS	Training set	0.74 (0.67‐0.81)	0.64 (0.58‐0.71)	0.93 (0.87‐1.00)	0.93 (0.86‐1.00)	0.75 (0.68‐0.82)	0.94 (0.88‐1.00)	0.96 (0.89‐1.00)
	Test set	0.59 (0.48‐0.70)	0.49 (0.37‐0.60)	0.61 (0.51‐0.72)	0.54 (0.44‐0.65)	0.64 (0.53‐0.75)	0.70 (0.59‐0.81)	0.73 (0.62‐0.84)
PFS	Training set	0.68 (0.62‐0.75)	0.63 (0.56‐0.69)	0.73 (0.66‐0.79)	0.80 (0.73‐0.87)	0.70 (0.63‐0.77)	0.81 (0.74‐0.88)	0.79 (0.72‐0.85)
	Test set	0.56 (0.45‐0.66)	0.50 (0.40‐0.61)	0.61 (0.50‐0.71)	0.45 (0.35‐0.56)	0.61 (0.51‐0.71)	0.68 (0.57‐0.78)	0.71 (0.60‐0.81)

Combining clinical and pathological variables (M5) did confer a non‐significant improvement (test‐set, p‐value: 0.18) above the performance of the clinical model (C‐index: development: 0.75 [0.68‐0.82], testing: 0.64 [0.53‐0.75]). The combination of all feature classes (M6) did trigger a further performance increase (C‐index: development: 0.94 [0.88‐1.00], testing: 0.70 [0.59‐0.81]) significantly better than all single feature class models alone (test‐set: M1 *P* = 0.018, M2 *P* = 0.003, M3 *P* = 0.044, M4 *P* = 0.003).

In the final model, treatment features describing the delivered therapeutic regimens were added to the model M7. This led to a further rise above the best performing combined model (M6) with a C‐index of 0.96 (0.89‐1.00) in the development set and 0.73 (0.62‐0.84) in the independent test set without reaching significance in direct comparison (*P* = 0.34).

The recently proposed nomogram from the work of Gittleman et al^26^ was tested on the independent test set using the 12‐month survival probabilities. It achieved a predictive performance with an AUC of 0.64 in comparison with AUCs of 0.75 and 0.80 for models M6 and M7.

**Figure 1:**
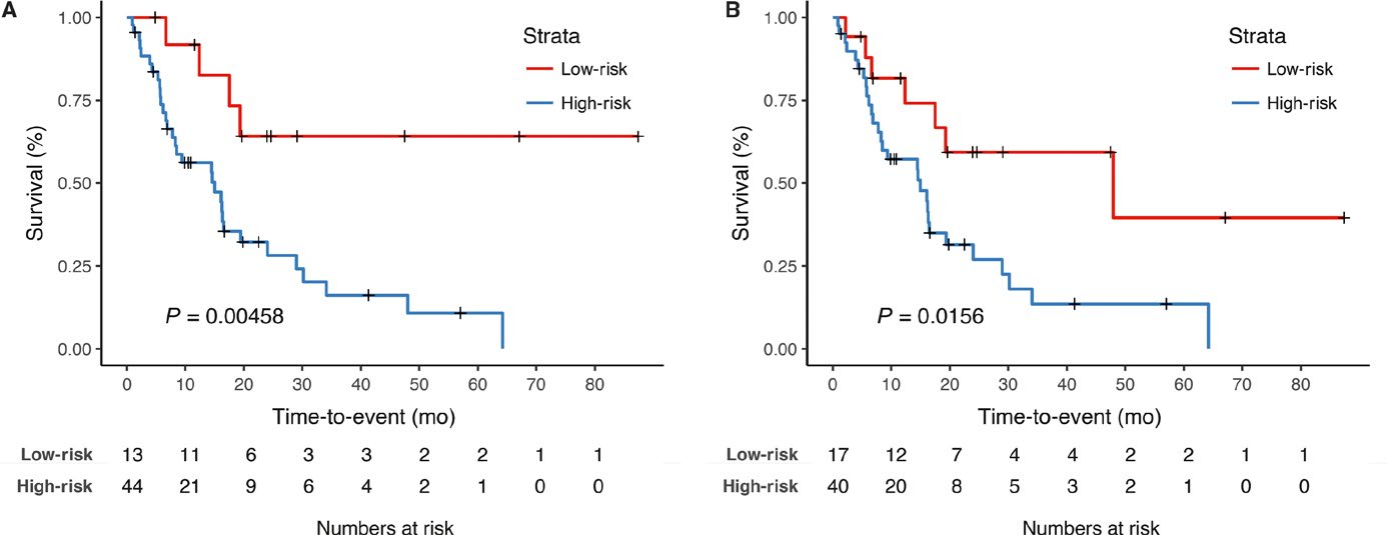
Kaplan‐Meier curves for overall survival showing the performance model 6 (M6) and model 7 (M7) in the internal validation cohort. The developed classifiers for overall survival M6 and M7 were used to assign patients to a “high‐risk” and “low‐risk” group in the validation patient cohort. The log‐rank test was applied to test for significant separation of survival curves and calculation of *P*‐values. Model 6 did divide significantly patient subgroups on the validation test set (*P* = 0.00458). Model 7 significantly divided high‐risk form low‐risk patients (*P* = 0.0156)

Next, two risk groups were defined by the predictors of the models M6 and M7 using cut‐off points optimized on the training set. Kaplan‐Meier survival curves of the validation cohort are shown in Figure 1. Both models significantly discerned high‐risk from low‐risk patients (M6 *P* = 0.0048, M7 *P* = 0.0156).

### 3.2 Prediction of patients' progression‐free survival

The same model categories were developed to predict PFS (see Table 4 for C‐index values and confidence intervals). Compared to the survival models, predictive performance was lower for the single feature class models clinical M1, pathological M2, MRI‐feature‐based M3, and FET‐PET/CT‐based M4 in the development set (C‐indices of 0.68, 0.63, 0.73, and 0.80, respectively). On the independent test set, prediction performance achieved even further reduced results (C‐indices 0.56, 0.50, 0.60, and 0.45 for M1, M2, M3, and M4, respectively).

The multimodal models predicted comparably to the best single feature class model M3. The combined clinical and pathological model 5 achieved a C‐index of 0.70 (0.63‐0.77) in the development and 0.61 (0.51‐0.71) in the independent test sets, which was non‐significantly compared to the clinical model M1 (test‐set, *P* = 0.096). The combined model M6 achieved C‐indices of 0.81 (0.74‐0.88) on the development set and 0.68 (0.57‐0.78) on the independent test set significantly outperforming the single feature class models M1 and M3 (M1 *P* = 0.016, M2 *P* = 0.196, M3 *P* = 0.014, M4 *P* = 0.233).

**Figure 2:**
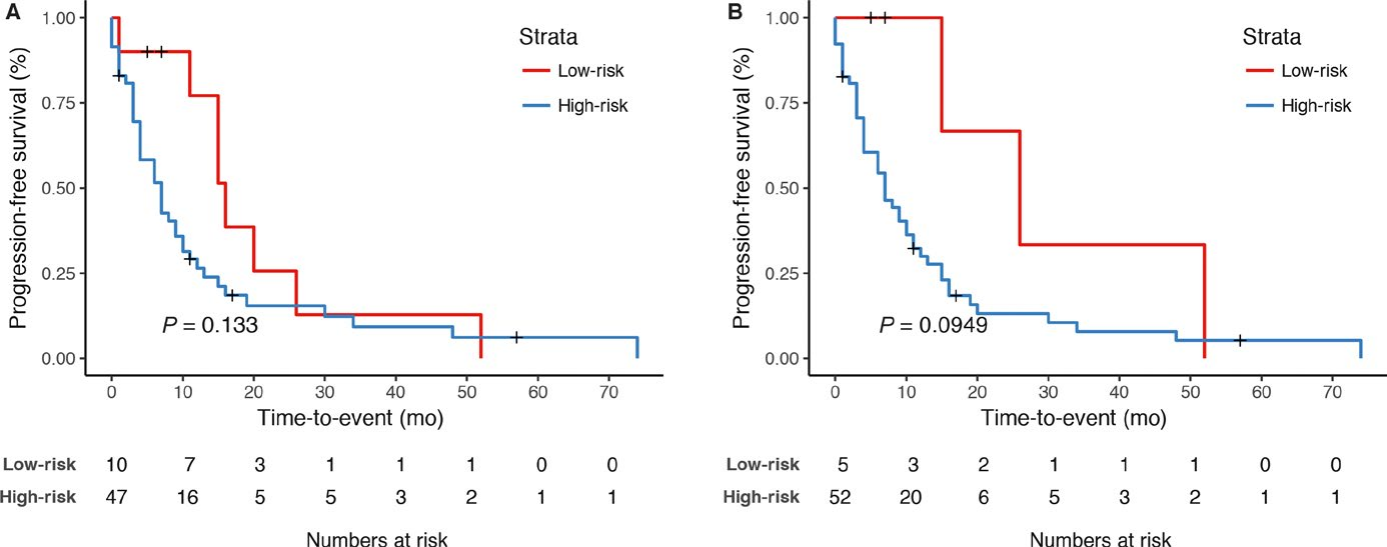
Kaplan‐Meier curves for progression‐free survival showing the performance of model 6 (M6) and model 7 (M7) in the internal validation cohort. The developed classifiers M6 and M7 for progression‐free survival were used to assign patients to a “high‐risk” and “low‐risk” group in the validation patient cohort. The log‐rank test was applied to test for significant separation of survival curves and calculation of *P*‐values. No significant separation of PFS curves could be observed for model 6 (*P* = 0.133). For model 7, there was a separation of survival curve without reaching statistical significance (*P* = 0.0949)

Finally, adding therapeutic information to M7 increased prognostic performance further up to a C‐index of 0.79 (0.72‐0.84) on the development set and 0.71 (0.60‐0.81) on the independent test set with a significantly better prediction than all other models (M1 *P* = 0.0013, M2 *P* = 0.023, M3 *P* = 0.00057, M4 *P* = 0.0001, M5 *P* = 0.002, M6 *P* < 0.0001). The Gittleman nomogram, which was generated to predict survival at 12 months, showed worse prognostic capacity with an AUC of 0.67 in comparison with AUCs of 0.82 and 0.83 for models M6 and M7. Kaplan‐Meier curves plotting PFS for patient subgroups separated by the prediction models did not show significant separations for M6 and M7 (see Figure 2). For M7, however, there was a trend toward significance (*P* = 0.095).

### 3.3 MRI‐based and therapy‐related feature dominate OS prediction performance

In order to evaluate the importance of single features for the performance of the combined models M6 and M7, the permutation feature importance was assessed (see Table S5). Consistent with the observation of model M3 as best single feature class model, MRI‐based features were the most important features besides patients' age. In contrast, model M7 was dominated by treatment features, such as single and total radiation dose, PTV volume and surgery. Besides, the feature classes MRI‐based, clinical, and pathological were among the 10 best performing features. The most important MRI‐based features in both models included "Satellites" and "Thickness of CE margin.".

In PFS prediction models, M6 and M7 MRI‐based features appeared to be the most important feature class beside the known prognostic factors "age," "KPS," "gender," and "MGMT" status (see Table S6). Model M7 showed a similar feature importance distribution as for OS with treatment features providing the most important features. The MRI‐based features "deep white matter invasion," "ependymal invasion," "proportion of resection of enhancing tumor," and of "edema" were among the 10 most important features in both models.

Consistent with the low performance of model M4, FET‐PET/CT‐based features did not show a high permutation importance for prediction of OS and PFS**.**

## 4 DISCUSSION

In this work, we have demonstrated the potential of ML‐based predictive models for the prognostic classification of GBM patients. Exploiting the capabilities of RF models to deal with large feature numbers and missing data, we assessed the value of certain feature classes and their combinatory effect. Moreover, we evaluated the benefit of integrating treatment features.

OS prediction was dominated by MRI‐based features having the highest single model performance (M3) and showing high feature representation in the combined models M6 and M7. The feature classes in clinical and pathological models showed lower predictive performance. However, clinical and pathological were consistently selected for the combined models M6 and M7. For both prediction tasks, combining all pre‐therapeutic feature classes inside model M6 did increase prognostic performance above the best single feature class model.

Finally, adding therapeutic information led to a further increase in prognostic performance for OS and PFS with a higher predictive performance compared to the formerly proposed nomogram by Gittleman et al.^26^ Kaplan‐Meier survival analysis showed a significant separation of high‐risk from low‐risk patients for the OS model.

Multiple previous studies have analyzed the prognostic potential of semantic MRI‐based features alone or in conjunction with clinical or pathological features. A simple model based only on three semantic imaging features "volume," "T1/FLAIR‐ratio," and "hemorrhage" achieved a 12‐month AUC of 0.67 for survival inferior to the combined models M6 and M7 (AUCs of 0.75 and 0.80, respectively) and similar to the clinical normogram by Gittleman et al^22^ Two further studies could demonstrate an incremental benefit by combining clinical features with VASARI features yielding a C‐index of 0.69, respectively.^20^, ^34^ In our study, model M6 combining MRI‐based features with clinical, pathological and FET PET/CT‐based features showed a similar performance of 0.69. This may indicate that clinical and semantic imaging may be sufficient for pre‐therapeutic prognostic assessment.

In recent years, quantitative computational imaging features ("radiomics") have been shown to add prognostic value above clinical and molecular factors in GBM patients.^35^, ^36^ Interestingly, a combined clinical and radiomic model achieved similar prognostic performances with C‐indices of 0.70 and 0.65 for OS and PFS, respectively. A radiomic model would have the great advantage of being less operator‐dependent. However, multiple technical hurdles including the dependency of image acquisition parameters, equipment, preprocessing, and feature extraction need to be solved before safe clinical applications. Until then, semantic imaging feature may constitute a valuable alternative that is less dependent on technical variances.

It should be noted that this study was performed on the basis of a retrospective patient cohort. However, treatment regimens were overall rather homogenous with primary therapy following the recommendation of Stupp et al^11^ in 74% of patients. Prognostic performance was tested on an internal validation cohort lowering available patients for model generation. In contrast to the above‐mentioned benefits of ML‐based models, model generation requires relatively large training sets. Limited patient numbers thus foster instability of model performances, which may further be increased by missing data.

In this study, we analyzed the predictive value of FET‐PET/CT‐based features in model M4. M4 showed high prognostic performances for both prediction tasks in the training set, which could not be reproduced in the independent test set. In contrast, previous studies have shown prognostic potential for OS and PFS.^23^, ^24^, ^25^ There are two reasons that might explain an underestimation of the FET‐PET/CT prognostic effect. First, the patient number with available PET data was relatively low compared to the total patient number. Second, two distinct reconstruction methods were used that may have led to inconsistencies in PET measures. A prospective study should be performed to evaluate the effect of FET‐PET/CT features. In the future, prognostic performance might be enhanced by including texture features or dynamic FET‐PET/CT measures.^25^, ^37^

Current prognostic models are often based on clinical information. In recent years, a large number of novel prognostic imaging and molecular‐based biomarkers have been identified.^38^, ^39^, ^40^, ^41^ Incorporating treatment features into a CDSS may increase the prognostic efficacy by quantifying the effect of partially given or omitted therapies.^27^

In summary, we demonstrated the applicability of ML models for the prediction of patients' OS and PFS. Semantic MRI‐based features for OS and PFS showed relevant prognostic value. The inclusion of treatment data further increased predictive performance and may help to optimize follow‐up procedures or 2nd line therapy regimens as CDSS.

## CONFLICT OF INTEREST

The authors declare that the research was conducted in the absence of any commercial or financial relationships that could be construed as a potential conflict of interest.
