# Supplementary material for: Combining multimodal imaging and treatment features improves machine learning‐based prognostic assessment in patients with glioblastoma multiforme
Source: Cancer Med. 2018 Dec 18;8(1):128–36. doi: 10.1002/cam4.1908 (PMC6346243; doi:10.1002/cam4.1908)
Supplement: Supplementary file 1 [file CAM4-8-128-s001.docx]

**Supplementary Data**

**Supplementary Table 1**

**MRI Acquisition parameters**

|  | TE (msec) | TR (msec) | Voxel spacing (mm) | Matrix | Flip angle |
| --- | --- | --- | --- | --- | --- |
| T2w | 80 | 3200 | 4x0.4x0.4 | 640x640 | 90 |
| (T1w) MPRAGE | 4 | 9 | 1x1x1 | 240x240 | 8 |
| 3D-FLAIR | 279 | 4800 | 1.05x1.05x1.05 | 240x240 | n/a |
| DWI | 55 | 7906 | 2x0.875x0.875 | 256x256 | 90 |

The following parameters were used for MRI image acquisition. For DWI-imaging b-values of 0 and 1000 were used. For contrast-enhancement, the contrast agent Magnograf (MaRoTrast, Jena, Germany) was administered intravenously (0.2 mL/kg, 0.5–1 mL/sec) with an MRI-compatible contrast medium injection system (Spectris Solaris EP; Siemens Medical, Erlangen, Germany) using a standardized protocol.

**Supplementary Table 2**

**Extracted VASARI features.**

| VASARI feature |
| --- |
| Pre-operative |
| Calvarial remodeling |
| CET crosses midline |
| Cortical involvment |
| Cysts |
| Deep wm invasion |
| Definition CE margin |
| Definition nCE margin |
| Diffusion |
| Edema crosses midline |
| Eloquent brain |
| Enhancement quality |
| Ependymal invasion |
| Hemorrhage |
| Multilocality |
| nCET crosses midline |
| Pial invasion |
| Proportion CET |
| Proportion edema |
| Proportion nCET |
| Proportion necrosis |
| Satellites |
| Size longest diameter |
| Size perpendicular diameter |
| Side of epicenter |
| T1/FLAIR RATIO |
| Thickness CE margin |
| Tumor location |
| Post-operative |
| Resection CET |
| Resection Edema |
| Resection nCET |

Features are listed in alphabetical order.

label: CE: contrast enhancing, CET: contrast enhancing tumor, FLAIR: fluid attenuated inversion recovery, nCE: non-contrast enhancing, nCET: non-contrast enhancing tumor, Perp: perpendicular, wm: white matter

**Supplementary Table 3**

**Regrouping of feature groups.**

| Feature | Change |
| --- | --- |
| Proportion necrosis | 2 groups: <34%; >34% |
| Proportion edema | 2 groups: <34%; >34% |
| Proportion nCET | 2 groups: <34%; >34% |
| Resection CET† | 2 groups: <95%; >95% |
| Resection nCET | 3 groups: <34%; 34%-95%; >95% |
| Resection edema | 3 groups: <34%; 34%-95%; >95% |
| Epicenter | combined: right + left |
| Thickness CE margin | combined: thin + none; thick + solid |
| Multilocality | combined: multilocal + multicentric |

**Supplementary Table 4**

**Results of FET-PET/CT features.**

|  | Median (minimum - maximum) |
| --- | --- |
| TBR mean | 2.0 (1.6 - 3.4) |
| TBR max | 3.2 (1.7 - 6.2) |
| MTV | 27468.2 (164.6 - 128071.9) |
| TLU | 55850.8 (270.5 - 311970.0) |

**Label:** MTV: mean tumor volume, TBR: tumor to brain ratio, TLU: total lesion uptake

**Supplementary Table 5 Importance assessment of the input features for survival models.**

The table shows feature permutation importance for the predictions of models 6 and 7 calculated on the patient test set.

| Model 6 |  | Model 7 |  |  |
| --- | --- | --- | --- | --- |
| Feature | **Importance** | **Feature** |  | **Importance** |
| Age | 2.93E-02 | Single dose |  | 3.10E-02 |
| Multilocality | 6.15E-03 | Total dose |  | 1.44E-02 |
| Satellites | 4.62E-03 | Age |  | 1.11E-02 |
| Proportion edema | 4.62E-03 | Satellites |  | 4.72E-03 |
| Edema crosses midline | 3.18E-03 | Surgery |  | 2.36E-03 |
| Deep wm invasion | 2.84E-03 | Multilocality |  | 2.35E-03 |
| Ependymal invasion | 2.69E-03 | PTV |  | 2.08E-03 |
| Side of Epicenter | 2.12E-03 | MGMT |  | 2.03E-03 |
| Thickness CE margin | 2.10E-03 | Thickness CE margin |  | 1.73E-03 |
| Size perpendicular diameter | 1.54E-03 | Tumor location occipital |  | 1.66E-03 |
| Tumor location occipital | 1.51E-03 | Ependymal invasion |  | 1.54E-03 |
| Cyst(s) | 1.34E-03 | Edema crosses midline |  | 1.51E-03 |
| KPS | 1.23E-03 | Size longest diameter |  | 1.13E-03 |
| TLU | 1.10E-03 | Deep wm invasion |  | 1.02E-03 |
| TBR max | 8.68E-04 | Resection nCET (<34%) |  | 9.03E-04 |
| Enhancement quality mild | 6.74E-04 | Ad. Ct |  | 4.95E-04 |
| Cortica involvment | 6.62E-04 | Definition nCE margin n.a |  | 4.36E-04 |
| Resection CET (34-95%) | 6.35E-04 | KI67 |  | 3.85E-04 |
| Resection edema (<34%) | 5.44E-04 | Side of Epicenter |  | 3.43E-04 |
| Eloquent brain speech motor | 5.27E-04 | MTV |  | 3.12E-04 |
| Resection nCET (>95%) | 5.15E-04 | Tumor location parietal |  | 2.80E-04 |
| Definition CE margin poorly defined | 5.10E-04 | Resection CET (<34%) |  | 2.79E-04 |
| Tumor location insular | 4.92E-04 | Calvarial remodeling |  | 2.78E-04 |
| Size longest diameter | 4.17E-04 | Tumor location frontal |  | 2.24E-04 |
| Definition nCE margin n.a | 4.09E-04 | Proportion edema |  | 2.22E-04 |
| Eloquent brain none | 3.75E-04 | RTCT |  | 1.95E-04 |
| Definition nCE margin smooth | 3.51E-04 | Enhancement quality marked |  | 1.93E-04 |
| Eloquent brain vision | 3.39E-04 | Eloquent brain speech motor |  | 1.79E-04 |
| Proportion necrosis | 2.83E-04 | CET crosses midline |  | 1.72E-04 |
| MGMT | 2.81E-04 | Tumor location insular |  | 1.71E-04 |
| CET crosses midline | 2.71E-04 | Proportion nCET |  | 1.43E-04 |
| Diffusion restricted | 2.60E-04 | Definition nCE margin smooth |  | 1.09E-04 |
| nCET crosses midline | 2.51E-04 | Gender |  | 9.07E-05 |
| Tumor location parietal | 2.45E-04 | Enhancement quality none |  | 4.27E-05 |
| Gender | 2.30E-04 | Enhancement quality mild |  | 1.91E-05 |
| Resection nCET (<34%) | 2.22E-04 | Diffusion neither.equal |  | 1.56E-05 |
| Diffusion neither.equal | 2.06E-04 | Proportion necrosis |  | 1.07E-05 |
| Calvarial remodeling | 2.05E-04 | Diffusion restricted |  | 9.79E-06 |
| Tumor location brainstem | 2.01E-04 | Definition CE margin n.a |  | -1.42E-05 |
| KI67 | 1.49E-04 | Proportion CET |  | -2.14E-05 |
| MTV | 8.72E-05 | TBR max |  | -2.36E-05 |
| Enhancement quality marked | 8.32E-05 | Tumor location brainstem |  | -4.09E-05 |
| Resection CET (<34%) | 7.12E-05 | nCET crosses midline |  | -6.58E-05 |
| IDH | 5.60E-05 | Tumor location temporal |  | -8.54E-05 |
| Proportion nCET | 1.25E-05 | Eloquent brain vision |  | -9.39E-05 |
| Enhancement quality none | -1.47E-05 | Resection edema (34-95%) |  | -1.09E-04 |
| Hemorrhage | -2.09E-05 | IDH |  | -1.29E-04 |
| Definition CE margin n.a | -3.51E-05 | Diffusion facilitated |  | -1.34E-04 |
| Resection edema (34-95%) | -9.16E-05 | TBR mean |  | -1.44E-04 |
| Resection nCET (34-95%) | -1.50E-04 | Eloquent brain speech recept |  | -1.57E-04 |
| Resection edema (>95%) | -1.84E-04 | T1.FLAIR RATIO infiltrative |  | -1.96E-04 |
| Diffusion facilitated | -1.96E-04 | Definition nCE margin irregeular |  | -1.97E-04 |
| TBR mean | -2.04E-04 | Resection nCET (34-95%) |  | -2.22E-04 |
| Tumor location temporal | -2.26E-04 | Eloquent brain none |  | -2.37E-04 |
| T1.FLAIR RATIO infiltrative | -2.69E-04 | Resection CET (34-95%) |  | -2.42E-04 |
| Resection CET (>95%) | -3.36E-04 | Cyst(s) |  | -2.48E-04 |
| Definition CE margin well defined | -3.72E-04 | Definition CE margin poorly defined |  | -2.60E-04 |
| Pial invasion | -3.76E-04 | Cortica involvment |  | -2.72E-04 |
| Eloquent brain speech recept | -4.55E-04 | KPS |  | -2.76E-04 |
| Definition nCE margin irregeular | -4.99E-04 | Pial invasion |  | -2.88E-04 |
| Proportion CET | -1.16E-03 | Size perpendicular diameter |  | -3.67E-04 |
| T1.FLAIR RATIO expansive | -1.19E-03 | Resection edema (<34%) |  | -3.71E-04 |
| Tumor location frontal | -1.26E-03 | Definition CE margin well defined |  | -3.85E-04 |
| Eloquent brain motor | -1.35E-03 | Resection edema (>95%) |  | -3.96E-04 |
| T1.FLAIR RATIO mixed | -1.38E-03 | Hemorrhage |  | -4.42E-04 |
|  | -2.15E-03 | Time .interval |  | -4.67E-04 |
|  |  | Resection nCET (>95%) |  | -5.82E-04 |
|  |  | Eloquent brain motor |  | -7.23E-04 |
|  |  | TLU |  | -8.14E-04 |
|  |  | Resection CET (>95%) |  | -1.01E-03 |
|  |  | T1.FLAIR RATIO expansive |  | -1.08E-03 |
|  |  | T1.FLAIR RATIO mixed |  | -1.72E-03 |

AUC: area under the receiver operating characteristic curve, adj: adjuvant, CET: contrast enhancing tumor, CT: chemotherapy, KPS: Karnofsky performance index, PTV: planning target volume, RT: radiation therapy, RTCT: radiochemotherapy, 2y: 2 year

**Supplementary Table 6: Importance assessment of the input features for progression models.**

The table shows feature permutation importance for the predictions of models 6 and 7 calculated on the patient test set.

| Model 6 |  | Model 7 |  |  |
| --- | --- | --- | --- | --- |
| Feature | **Importance** | **Feature** |  | **Importance** |
| Deep wm invasion | 1.10E-02 | Total dose |  | 1.65E-02 |
| MGMT | 1.10E-02 | Single dose |  | 1.46E-02 |
| Age | 7.72E-03 | MGMT |  | 1.03E-02 |
| KPS | 4.18E-03 | Deep wm invasion |  | 8.17E-03 |
| Resection CET (34-95%) | 3.57E-03 | PTV |  | 5.68E-03 |
| Resection CET (>95%) | 2.26E-03 | Age |  | 3.24E-03 |
| Multilocality | 1.94E-03 | KPS |  | 2.48E-03 |
| Gender | 1.14E-03 | Ependymal invasion |  | 2.24E-03 |
| Resection edema (<34%) | 9.84E-04 | Resection CET (34-95%) |  | 1.42E-03 |
| Ependymal invasion | 8.59E-04 | Resection edema (<34%) |  | 1.19E-03 |
| Tumor location temporal | 8.47E-04 | Size perpendicular diameter |  | 1.19E-03 |
| Satellites | 8.28E-04 | Resection CET (>95%) |  | 1.04E-03 |
| Resection nCET (>95%) | 8.23E-04 | Gender |  | 9.59E-04 |
| Definition CE margin poorly defined | 8.11E-04 | Multilocality |  | 9.05E-04 |
| Calvarial remodeling | 7.05E-04 | Surgery |  | 8.71E-04 |
| Thickness CE margin | 6.11E-04 | Tumor location temporal |  | 8.28E-04 |
| Tumor location occipital | 5.96E-04 | Tumor location occipital |  | 7.25E-04 |
| Pial invasion | 4.38E-04 | Resection edema (34-95%) |  | 5.28E-04 |
| Size perpendicular diameter | 4.21E-04 | Resection nCET (>95%) |  | 5.04E-04 |
| T1.FLAIR RATIO expansive | 4.11E-04 | Resection nCET (34-95%) |  | 4.19E-04 |
| Definition nCE margin n.a | 3.64E-04 | Satellites |  | 4.06E-04 |
| Diffusion neither.equal | 3.35E-04 | Pial invasion |  | 3.54E-04 |
| Diffusion restricted | 3.35E-04 | Size longest diameter |  | 3.37E-04 |
| Resection edema (34-95%) | 3.28E-04 | Definition CE margin poorly defined |  | 3.17E-04 |
| Side of Epicenter | 3.03E-04 | Calvarial remodeling |  | 3.10E-04 |
| T1.FLAIR RATIO infiltrative | 3.01E-04 | Thickness CE margin |  | 2.50E-04 |
| Eloquent brain speech recept | 2.98E-04 | Diffusion restricted |  | 2.44E-04 |
| nCET crosses midline | 2.90E-04 | Enhancement quality mild |  | 2.30E-04 |
| Resection edema (>95%) | 2.44E-04 | Definition nCE margin n.a |  | 2.26E-04 |
| Resection nCET (34-95%) | 2.20E-04 | Cyst(s) |  | 2.16E-04 |
| CET crosses midline | 1.17E-04 | MTV |  | 2.04E-04 |
| Definition CE margin n.a | 6.90E-05 | Enhancement quality marked |  | 1.96E-04 |
| MTV | 6.01E-05 | Eloquent brain speech recept |  | 1.69E-04 |
| Enhancement quality none | 5.76E-05 | T1.FLAIR RATIO infiltrative |  | 1.65E-04 |
| Proportion nCET | 5.51E-05 | Tumor location parietal |  | 1.57E-04 |
| Cortica involvment | 3.54E-05 | Resection edema (>95%) |  | 1.44E-04 |
| Definition CE margin well defined | 3.40E-05 | Enhancement quality none |  | 1.36E-04 |
| Eloquent brain vision | 6.80E-06 | Definition nCE margin smooth |  | 1.27E-04 |
| Proportion CET | -5.19E-05 | CET crosses midline |  | 1.18E-04 |
| KI67 | -5.29E-05 | T1.FLAIR RATIO expansive |  | 1.18E-04 |
| TBR max | -6.15E-05 | Edema crosses midline |  | 1.08E-04 |
| Size longest diameter | -6.80E-05 | Proportion nCET |  | 9.66E-05 |
| Cyst(s) | -7.62E-05 | RTCT |  | 9.33E-05 |
| Tumor location parietal | -1.04E-04 | Definition CE margin well defined |  | 8.76E-05 |
| IDH | -1.24E-04 | KI67 |  | 7.19E-05 |
| Proportion edema | -1.39E-04 | Cortica involvment |  | 2.43E-05 |
| TBR mean | -1.90E-04 | IDH |  | 2.32E-05 |
| Resection CET (<34%) | -2.26E-04 | Definition CE margin n.a |  | 2.07E-05 |
| Edema crosses midline | -2.39E-04 | Eloquent brain none |  | 4.65E-06 |
| Enhancement quality marked | -2.61E-04 | Diffusion facilitated |  | 3.93E-06 |
| T1.FLAIR RATIO mixed | -2.95E-04 | Side of Epicenter |  | 3.58E-07 |
| Resection nCET (<34%) | -3.15E-04 | Eloquent brain speech motor |  | -2.86E-06 |
| Diffusion facilitated | -3.15E-04 | Resection CET (<34%) |  | -3.72E-05 |
| Eloquent brain none | -3.30E-04 | Diffusion neither.equal |  | -4.40E-05 |
| Eloquent brain motor | -3.74E-04 | TLU |  | -5.11E-05 |
| Enhancement quality mild | -4.03E-04 | T1.FLAIR RATIO mixed |  | -5.51E-05 |
| Eloquent brain speech motor | -4.62E-04 | nCET crosses midline |  | -6.65E-05 |
| Tumor location brainstem | -4.67E-04 | TBR max |  | -7.37E-05 |
| Hemorrhage | -6.02E-04 | Time .interval |  | -1.09E-04 |
| Proportion necrosis | -6.14E-04 | Proportion CET |  | -1.23E-04 |
| TLU | -6.21E-04 | Resection nCET (<34%) |  | -1.23E-04 |
| Tumor location frontal | -6.69E-04 | Eloquent brain vision |  | -1.46E-04 |
| Tumor location insular | -7.79E-04 | Proportion edema |  | -1.55E-04 |
| Definition nCE margin smooth | -8.32E-04 | Proportion necrosis |  | -2.07E-04 |
| Definition nCE margin irregular | -1.89E-03 | Tumor location brainstem |  | -2.11E-04 |
|  |  | Eloquent brain motor |  | -2.50E-04 |
|  |  | Tumor location insular |  | -2.65E-04 |
|  |  | TBR mean |  | -3.23E-04 |
|  |  | Ad. Ct |  | -3.78E-04 |
|  |  | Hemorrhage |  | -3.78E-04 |
|  |  | Tumor location frontal |  | -7.59E-04 |
|  |  | Definition nCE margin irregular |  | -9.66E-04 |

label: 2y: 2 year**,** AUC: area under the receiver operating characteristic curve, adj: adjuvant, CET: contrast enhancing tumor, CT: chemotherapy, KPS: Karnofsky performance index, MTV: metabolic target volume, TBR: tumor to brain ratio, nCET: non contrast enhancing tumor, PTV: planning target volume, RT: radiation therapy, RTCT: radiochemotherapy, TLU: total lesion uptake
